# Metabolomics of Small Intestine Neuroendocrine Tumors and Related Hepatic Metastases

**DOI:** 10.3390/metabo9120300

**Published:** 2019-12-11

**Authors:** Alessio Imperiale, Gilles Poncet, Pietro Addeo, Elisa Ruhland, Colette Roche, Stephanie Battini, A. Ercument Cicek, Marie Pierrette Chenard, Valérie Hervieu, Bernard Goichot, Philippe Bachellier, Thomas Walter, Izzie Jacques Namer

**Affiliations:** 1 Biophysics and Nuclear Medicine, University Hospitals of Strasbourg, 67098 Strasbourg, France; elisa.ruhland@chru-strasbourg.fr (E.R.); izziejacques.namer@chru-strasbourg.fr (I.J.N.); 2 Faculty of Medicine, University of Strasbourg, FMTS, 67000 Strasbourg, France; marie-pierrette.chenard@chru-strasbourg.fr (M.P.C.); bernard.goichot@chru-strasbourg.fr (B.G.); philippe.bachellier@chru-strasbourg.fr (P.B.); 3 MNMS Platform, University Hospitals of Strasbourg, 67098 Strasbourg, France; stephanie.battini@yahoo.com; 4 Molecular Imaging—Institut Pluridisciplinaire Hubert Curien (IPHC), UMR 7178 – CNRS/Unistra, 67098 Strasbourg, France; 5 Digestive and Oncologic Surgery, Edouard-Herriot University Hospital, Claude-Bernard Lyon 1 University, 69622 Lyon, France; gilles.poncet@chu-lyon.fr; 6 Hepato-Pancreato-Biliary Surgery and Liver transplantation, University Hospitals of Strasbourg, University of Strasbourg, 67098 Strasbourg, France; pietrofrancesco.addeo@chru-strasbourg.fr; 7 INSERM U1052/CNRS UMR5286/University of Lyon, Cancer Research Center of Lyon, 69622 Lyon, France; colette.roche@inserm.fr (C.R.); valerie.hervieu@chu-lyon.fr (V.H.); 8 Computer Engineering Department, Bilkent University, Ankara 06800, Turkey; cicek@cs.bilkent.edu.tr; 9 Pathology, University Hospitals of Strasbourg, Strasbourg University, 67098 Strasbourg, France; 10 Tissu-Tumorothèque Est (CRB-HCL, Hospices Civils de Lyon Biobank, BB-0033-00046), 69622 Lyon, France; 11 Internal Medicine, Diabetes and Metabolic Disorders, University Hospitals of Strasbourg, Strasbourg University, 67098 Strasbourg, France; 12 Medical Oncology, Edouard Herriot Hospital, Hospices Civils de Lyon, 69622 Lyon, France; thomas.walter@chu-lyon.fr; 13 University of Lyon, Université Lyon 1, 69622 Lyon, France

**Keywords:** neuroendocrine tumors, carcinoid, metabolomics, spectroscopy, NMR

## Abstract

To assess the metabolomic fingerprint of small intestine neuroendocrine tumors (SI-NETs) and related hepatic metastases, and to investigate the influence of the hepatic environment on SI-NETs metabolome. Ninety-four tissue samples, including 46 SI-NETs, 18 hepatic NET metastases and 30 normal SI and liver samples, were analyzed using 1H-magic angle spinning (HRMAS) NMR nuclear magnetic resonance (NMR) spectroscopy. Twenty-seven metabolites were identified and quantified. Differences between primary NETs vs. normal SI and primary NETs vs. hepatic metastases, were assessed. Network analysis was performed according to several clinical and pathological features. Succinate, glutathion, taurine, myoinositol and glycerophosphocholine characterized NETs. Normal SI specimens showed higher levels of alanine, creatine, ethanolamine and aspartate. PLS-DA revealed a continuum-like distribution among normal SI, G1-SI-NETs and G2-SI-NETs. The G2-SI-NET distribution was closer and clearly separated from normal SI tissue. Lower concentration of glucose, serine and glycine, and increased levels of choline-containing compounds, taurine, lactate and alanine, were found in SI-NETs with more aggressive tumors. Higher abundance of acetate, succinate, choline, phosphocholine, taurine, lactate and aspartate discriminated liver metastases from normal hepatic parenchyma. Higher levels of alanine, ethanolamine, glycerophosphocholine and glucose was found in hepatic metastases than in primary SI-NETs. The present work gives for the first time a snapshot of the metabolomic characteristics of SI-NETs, suggesting the existence of complex metabolic reality, maybe characteristic of different tumor evolution.

## 1. Introduction

Small-intestine (SI) neuroendocrine tumors (NETs) are the most common malignant NETs. SI-NETs are usually small, multiple in about 30%–50% of cases, and associated with lymph node and liver metastases at diagnosis in more than 90% and 50% of patients, respectively. SI-NETs are slow-growing lesions and are associated with a carcinoid syndrome in 20% of cases [1,2,3]. World Health Organization (WHO) classification is related to their cellular differentiation (poorly or well differentiated) and proliferation rate (grades 1–3) reflecting the degree of tumor biological aggressiveness [4]. Well-differentiated NETs constitute a very heterogeneous group, including low-grade tumors that are slowly progressive but able to lead to metastasis or recurrence after treatment, and more aggressive tumors with a rapid rate of progression. Faced with this great diversity of biological behavior, to predict tumor evolution may be very challenging. The presence of metastases at the time of diagnosis confirms the aggressive nature of the tumor, but a variable degree of disease progression can be observed. In patients with no metastatic NETs, it is essential to identify markers able to predict tumor progression; one possible approach is to define the metabolic fingerprint of the tumor compared to normal tissue.

Metabolism of cancer cells is extremely different from that of normal cells. The former take up large amounts of glucose that will be metabolized into lactate, even in the presence of oxygen [5]. In tumors, several metabolic intermediates are often generated from alternative metabolic pathways that could be driven by mutations or epigenetic alterations in oncogenes or tumor suppressor genes. Moreover, the extracellular microenvironment, hypoxia, metabolic restriction and tissue-specific signaling, also influence tumor metabolism and development. The understanding of metabolic pathways that are modified and reprogrammed in cancer cells can be potentially used for the development of novel therapeutic options targeting critical processes for tumor growth, as the metabolism of nucleotides, amino acids, carbon and fatty acids [6,7,8].

Simultaneous detection of multiple metabolites, also known as metabolomics, is a growing field that promises to improve the knowledge of cell biology, also linking genetics and epigenetics to tumor phenotypes [9]. In recent years, high-resolution 1H magic angle spinning (HRMAS) nuclear magnetic resonance (NMR) NMR spectroscopy has been proposed and successfully used for the ex vivo characterization of small intact tissue samples [10,11]. The simplicity of sample preparation, the intra- and inter-laboratory reproducibility [12], the relatively low cost (less than $50 per sample) and the availability of exhaustive metabolite databases [13,14], are significant advantages of HRMAS NMR spectroscopy.

A typical statistical approach to explore metabolomic data sets includes multivariate analysis methods such as partial least squares discriminant analysis (PLS-DA), allowing the identification of spectral characteristics that contribute most to the variation or separation of samples for further analysis [15,16,17]. The algorithm to determine expected metabolite level alterations (ADEMA) is an interesting alternative to manage metabolomic data. ADEMA is able to evaluate the changes in groups of metabolites between cases and controls instead of analyzing metabolites one by one, and uses mutual information to predict the expected change in direction per metabolite. ADEMA minimizes the loss of biological information related to a metabolic profile, especially when a disease causes slight changes in metabolite concentration that are insignificant at a single metabolite level, but significant when considered together with other metabolite levels.

Accordingly, ADEMA is able to determine activity rates for pathways based on changes due to disease and may be successfully used for network analysis based on metabolomic data [18].

In the present study, we first assessed the metabolomic fingerprint of primary SI-NETs using HRMAS NMR spectroscopy in searching for biomarkers related to clinical parameters and tumor pathological features. Finally, we compared the metabolome of primary SI-NETs with that of NET liver metastases in order to evaluate the influence of the environment on primary tumor metabolism.

## 2. Results

### 2.1. Patient Population and Tumor Characteristics

Among the 46 selected patients, there were 28 men (60%) and 18 women with a mean age of 61 years (range, 38–82 years). Thirteen patients (29%) presented with carcinoid syndrome. According to pathology and imaging results, 31 patients (67%) were considered to have systemic metastatic disease (liver, bone metastases and/or peritoneal carcinomatosis). All SI-NETs come from the ileum and 16 patients (35%) had multiple tumors. The median ki-67 index of primary SI-NETs was 2% (range, 1–13%). According to the 2010 WHO classification [3], tumors were classified as G1 and G2 in 35 and 11 patients, respectively. There were no G3 tumors. Perineural invasion and angioinvasion were observed in 13 of 21 and eight of 24 SI-NET patients, respectively. In the remaining cases, pathological data on perineural and angioinvasion were missing. Data on parietal infiltration were available from 45 tumor specimens showing tumor invasion of the muscle layer and serosa in 40 cases; tumor infiltration was limited to mucosa and submucosa in the remaining five NETs. Finally, the 18 specimens of liver metastases from 13 patients were graded as G1 and G2 in 14 and four cases, respectively.

### 2.2. HRMAS NMR Spectra

**Figure 1:**
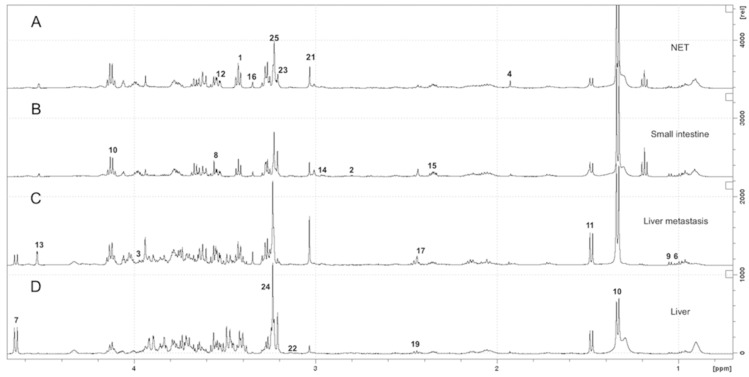
Representative 1D 1H Carr-Purcell-Meiboom-Gill (CPMG) high-resolution 1H magic angle spinning (HRMAS) spectra between 4.7 and 0.70 ppm of G2 neuroendocrine tumor (NET) (**A**), normal small-intestine (**B**), hepatic NET metastasis (**C**), and normal liver parenchyma (**D**).

At visual inspection, all the spectra obtained from the 94 tissue samples analyzed, comprising 46 SI-NETs, 18 hepatic NET metastases and 30 normal SI tissue and liver parenchyma, were good quality and were considered for the analyses. The representative 1D HRMAS Carr-Purcell-Meiboom-Gill (CPMG) spectra of G1 and G2 SI-NET, normal SI, hepatic NET metastasis and normal liver parenchyma, are presented in Figure 1.

The region between 7.50 and 0.70 ppm of each spectrum was analyzed. The partial metabolite assignment is indicated. The numbers refer to the metabolites listed in Table S1 (Supplementary Data). The metabolic content of healthy and cancerous tissue can be directly compared since the intensity of each spectrum was normalized with respect to the weight of the sample.

Within the range of 7.50–0.70 ppm, the following 27 metabolites were identified and quantified corresponding to well-defined spectral peaks with no major mutual overlapping: taurine, aspartate, serine, acetate, N-acetyl aspartate (NAA), isoleucine, glucose, glycine, valine, lactate, alanine, myo-inositol, ascorbate, glutathione (GSH), glutamate, scyllo-inositol, succinate, fumarate, glutamine, arginine, creatine, ethanolamine, choline, glycerophosphocholine, phosphorylcholine, tyrosine and tryptophan.

### 2.3. PLS-DA Results

**Figure 2:**
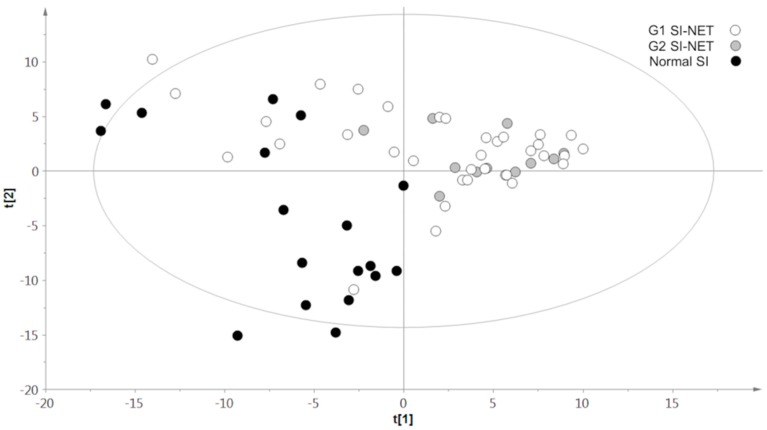
Results of three-class partial least squares discriminant analysis (PLS-DA) models performed including grade 1 (G1) neuroendocrine tumors (NETs) (white circles), grade 2 (G2) NETs (gray circles), and normal small-intestine tissue (black circles).

The generated multivariate two-component PLS-DA model performed on NETs and normal SI allowed partial separation of the two groups of samples. Nonetheless, acceptable cumulative confidence criterion of fit (R2Y:0.6) and prediction (Q2:0.5) were obtained. Succinate, glutathion (GSH), taurine, myoinositol and glycerophosphocholine were characteristic and more abundant in NET samples. On the other hand, higher levels of alanine, creatine, ethanolamine and aspartate were found in normal SI tissues. A two-component three-class PLS-DA revealed a continuum-like distribution among normal SI, G1-SI-NETs and G2-SI-NETs. The G2-SI-NET distribution was closer and clearly separated from normal SI tissue. On the other hand, G1-SI-NETs showed a wide spread and substantial overlap with samples of G2-SI-NETs and normal SI (R2Y:0.3; Q2:0.2). Further independent, two-class PLS-DA models, including normal SI tissue and G1 (R2Y:0.6; Q2:0.4) or G2 tumors (R2Y:0.8; Q2:0.8), confirmed these findings (Figure 2).

A two-component PLS-DA based on liver metastases and normal hepatic parenchyma was characterized by a good representation of the Y data (R2Y:0.9) and a good cumulative confidence criterion of prediction (Q2:0.9). A higher abundance of acetate, succinate, choline, phosphocholine, taurine, lactate and aspartate was found to discriminate liver metastases from normal liver. On the other hand, increased quantities of glucose, alanine, ethanolamine and valine characterized samples of healthy hepatic parenchyma. The generated multivariate two-component PLS-DA model built on primary SI-NETs and liver metastases allowed incomplete separation of the two groups of samples, showing partial overlap between the two classes (R2Y:0.5; Q2:0.4).

**Figure 3:**
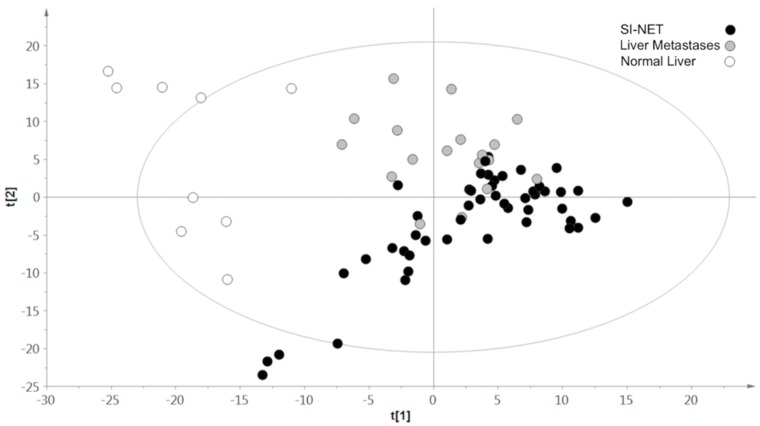
Results of three-class partial least squares discriminant analysis (PLS-DA) models including primary ileal NETs (black circles), hepatic NET metastases (gray circles), and normal liver parenchyma (white circles).

The amount of succinate, valine and myoinositol was higher in primary SI-NETs than metastases. In contrast, a higher level of alanine, ethanolamine, glycerophosphocholine and glucose was found in hepatic lesions. A three-component, three-class PLS-DA built on primary SI-NETs, liver NET metastases and normal liver parenchyma provided an overall representation of the data, confirming the clear separation of tumor samples from normal tissue, but a certain overlap of SI-NETs and hepatic metastases (R2Y:0.4; Q2:0.4) (Figure 3).

No statistically significant correlation was found between the preoperative serum chromogranin A level and the tissue metabolic profile. No other biological marker collected before surgery was available for further comparisons with tissue metabolic profile.

### 2.4. Univariate and ADEMA Network Analysis

The Mann Whitney U test showed significantly higher values of isoleucine (*p*:0.039), valine (*p*:0.028) and ethanolamine (*p*:0.041) in G1 than G2 tumors. No other discriminant metabolites were found using the Mann-Whitney U test according to the remaining clinical and pathological variables.

Seven independent ADEMA models were performed to relate metabolite changes to: patient metastatic status, tumor secretion, primary tumor multifocality, tumor grade, primary tumor perineural invasion and angioinvasion and the degree of primary tumor parietal infiltration. The ADEMA results are detailed in Table 1 showing the predicted changes in direction per metabolite.

For almost all of these models, the Mann-Whitney U test did not yield any significant differences, whereas ADEMA reports network metabolic changes. Thus, ADEMA appears more effective, as it compares different groups of metabolites predicting the expected change, uses mutual information and does not require a large population of samples to determine whether some metabolites are biomarkers when considered together.

**Figure 4:**
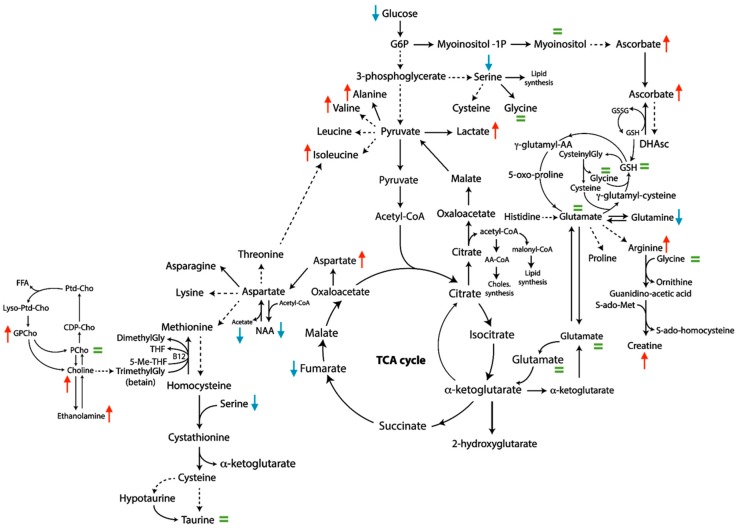
Metabolic subnetwork showing the results of ADEMA analysis including metastatic and nonmetastatic patients at diagnosis. Red arrows show metabolites that are predicted to increase, blue arrows show metabolites that are predicted to decrease; the remaining metabolites are expected to remain the same (green =).

Focusing on the clinical variable: “metastatic status at diagnosis”, 46 samples of SI-NETs from 31 metastatic patients and 15 patients without metastatic spread were included in the analysis. NETs from metastatic patients showed an increased level of choline, glycerophosphocholine, ethanolamine, aspartate, tryptophan, isoleucine, valine, alanine, lactate, ascorbate, arginine and creatine, as well as a decreased level of serine, acetate, NAA, fumarate, tyrosine, glucose, serine and glutamine. The other metabolites were predicted to be equivalent between the two groups (Figure 4).

## 3. Discussion

In the present study, using HRMAS NMR spectroscopy, we have provided the metabolomic profile of primary SI-NETs, starting from the analysis of snap-frozen tumor samples out of a population of 46 patients. Tumor metabolome was afterwards related to several pathological features, and patient clinical parameters have also been identified. Finally, we have reported the modification of the primary SI-NET metabolome after liver metastatic colonization.

The metabolome of gastroenteropancreatic (GEP) NETs has been explored by very few authors. In 2013, Kinross et al. were the first to apply a 1H NMR technique to analyze urine from a prospective cohort of 28 patients with GEP NETs (including only eight SI-NETs) providing potential tumor biomarkers [19]. Unfortunately, because of the retrospective character of our study, a comparison between the metabolic profile of tissular specimen and patient plasma or urine samples is not possible. However, despite methodological differences preventing direct and definitive comparison, creatine was lower in NET samples compared with healthy controls in both ours and the study of Kinross et al. [19]. In our population, no significant correlation was found between the preoperative serum chromogranin A level and the tissue metabolic profile.

PLS-DA models performed including G1 NETs, G2 NETs and normal small-intestine tissue revealed a continuum-like distribution of samples from normal SI to G2 NETs, suggesting increasing malignant potential encompassing the pathological concept of tumor grade.

Moreover, the distribution of G1 NETs was larger than that of G2 NETs, probably reflecting tumor biological heterogeneity. Metabolites produced by cancer cells mirror variations of normal metabolic pathways. The NET metabolic profile obtained from our patients suggests alterations of metabolic pathways that are crucial for cell proliferation such as the tricarboxylic acid cycle (TCA cycle). It is well known that succinate is an important metabolite implicated in numerous metabolic pathways (typically the TCA cycle), and it is involved in the production and removal of reactive oxygen species (ROS), as well as contributing to carcinogenesis. Germline and somatic mutations in enzymes implicated in the TCA cycle, as the succinate dehydrogenase (SDH), have been identified in several diseases as paragangliomas, contributing to defining the role of succinate as the metabolic hallmark in cancer. These mutations cause dysfunction of the TCA cycle and accumulation of metabolic intermediates, modifying the function of cancer cells [20,21]. Recently it has been shown that intratumoral injection of succinate significantly increases glucose consumption by endothelial cells of connective tissues in a tumor small-animal model [22], highlighting the complex role of succinate that encompasses epigenetics, tumorigenesis, signaling transduction, as well as endocrine and paracrine modulation [23]. Besides succinate, NETs showed an up-regulation of choline-containing compound metabolism, suggesting either accumulation or excess depletion of these metabolites for aberrant cell membrane turnover. Moreover, NETs were distinguished from normal SI tissue because of higher levels of GSH and taurine. GSH synthesis is inducted as a response to the higher oxidative stress. Its high redox potential renders GSH a potent antioxidant determinant in maintaining cellular redox balance. Taurine is a semi-essential amino acid that is important as an osmolyte, antioxidant and free-radical scavenger that protects cells against oxidative damage. It also inactivates hypochlorous acid, a strong cytotoxic oxidant, by forming a stable complex, which down-regulates immune responses leading to tumor development [24,25]. Higher levels of taurine have also been reported in the literature for several types of cancer [16], probably due to increased apoptosis. Finally, increased myoinositol levels contribute to the metabolomic fingerprint of NETs. Myoinositol is the precursor of phosphatidylinositol, a constituent of phospholipid membranes. It is involved in cell signaling and glucose and lipid metabolism. Myoinositol depletion affects cell survival and growth [26,27]. A germline deletion in the gene inositol polyphosphate multikinase (IPMK), with consequently reduced kinase activity, a reduced activation of p53 and increased cell survival, were identified by linkage analysis and whole-exome sequencing in familial cases of SI-NETs [28]. In our cohort, multiple SI-NETs presented a higher tissue concentration of myoinositol and scylloinositol, rather than solitary tumors. More investigations are necessary, however, to establish a causal link necessary to use inositol-containing compounds as biomarkers of tumor multifocality.

Whole metabolic profile and differences in single metabolite concentration have been assessed according to patient metastatic status, tumor secretion, primary tumor multifocality, tumor grade, primary tumor perineural invasion and angioinvasion and the degree of primary tumor parietal infiltration. In these cases, the Mann-Whitney U test did not yield any significant differences, whereas ADEMA reports some metabolic changes between groups. ADEMA appears more effective to determine whether those metabolites are biomarkers when considered together, and it can predict the expected change, as it compares different groups of metabolites, uses mutual information and does not require a large population of samples. Thus, According to ADEMA results (Table 1), the metabolic fingerprint of NETs with more pronounced pathological and clinical characteristics of aggressiveness, was characterized by reduced levels of glucose and a high concentration of lactate, suggesting an increased glycolytic rate. These findings support the use of ^18^F-radiolabeled deoxyglucose (^18^F-FDG) as a molecular probe for positron emission tomography (PET) investigation in patients with aggressive SI-NETs, supporting the evidence that tumor-increased glucose metabolism reveals worse patient prognoses [29]. High levels of alanine have been found in metastatic tumors and lesions with deep parietal invasion. The increased rate of protein degradation related to cell death or the deregulation of the Krebs cycle may explain the observed, altered amino acid level. Concentrations of serine and glycine were reduced in samples of aggressive tumor. Serine metabolism is frequently deregulated in cancer cells, and its importance has been actively investigated [30].

Exogenous serine is the third most quickly utilized nutrient by cultured cancer cells [31,32]. Serine can be converted to glycine, donating a one-carbon unit to the folate pool for nucleotide biosynthesis. It seems that rapidly proliferating cells switch to glycine utilization only after exhausting exogenous serine. This may explain why glycine depletion strongly correlates with the proliferation rate in cancer cells [31,32].

Liver metastatic colonization is a real problem in the management of SI-NETs, and patients often show voluminous liver metastases and a very small primary tumor. The microenvironment influences cancer cell metabolism, which could be altered by the macromolecules present in the extracellular compartment and by the secretion of tumor products related to its high metabolic rate. Typically, glucose-avid cancer cells alter the tumor microenvironment by secreting lactate that directly affects the behavior of stromal cells [33,34]. Liver NET metastases showed higher quantities of typical biomarkers of malignancy, such as choline, phosphocholine, taurine and lactate. Acetate, succinate and aspartate also contribute to define the metabolic fingerprint of liver metastases. Acetate could be considered as an index of fatty acid synthesis following beta-oxidation of acetyl coenzyme A (acetyl CoA) formed by the esterification of acetate. Aspartate is crucial for cellular functioning, and it has been shown that deficiency of aspartate synthesis is responsible for significant reduction of cancer cell proliferation when respiration is impaired. Considering that cancer cells lack asparaginase activity to convert asparagine to aspartate and that aspartate has poor cell permeability preventing exogenous intake, therapeutic aspartate suppression could be useful to treat cancer [35].

Glucose, alanine and ethanolamine were the discriminant metabolites of liver metastases when matched to primary SI-NETs. Interestingly, increased quantities of glucose, alanine, valine and ethanolamine were detected by HRMAS NMR spectroscopy in normal liver compared to liver metastases underlying the influence of liver tissue, which confers to metastases singular metabolic characteristics related to the intimate biology of the colonized organ. Our findings confirm that in metastatic cells a distinct metabolic reprogramming occurs depending on the site of metastasis [36].

Despite its main speculative design preventing definitive causal conclusions or therapeutic implications, the present work gives, for the first time, a snapshot of metabolomic characteristics of SI-NETs using ex vivo NMR spectroscopy. Moreover, we provide new data concerning the influence of the environment on the tumor metabolic profile in metastatic liver disease. However, the relatively small number of tissue samples and the lack of a prior hypothesis make this study a preliminary investigation. The real clinical impact of these results should be assessed by longitudinal studies evaluating patient survival in order to detect reliable metabolic fingerprints predictive of rapid tumor progression. The limited number of aggressive G2-NETs and the absence of G3 tumors are additional limitations of the present study. However, medical treatment is generally preferred to surgery in these cases, justifying the lack of pathological specimens obtained before chemotherapy, if any. In addition, depending on the surgical technique, there is a variable amount of intraoperative tissue ischemia due to surgical ligation of blood vessels with potential consequences on the tissular metabolic profile. Thus, a standardized protocol is necessary to increase reproducibility of data. Although the metabolic profile can be altered by tissue freezing, there is often no better alternative because there is no logistics for direct analysis of fresh tissue after surgery, hence the need to freeze the tissue and store it at −80°. In addition, data indicate that the long-term, frozen storage of tissue samples can be quantitatively analyzed by HRMAS MRS without significant degradation or alteration of metabolic profile [37].

## 4. Materials and Methods

### 4.1. Patient Population

For the present study, 46 tissue specimens of ileal neuroendocrine tumors (NETs) obtained from 46 patients who had undergone surgery were retrospectively selected according to the following criteria: (1) final diagnosis according to the pathological standards, (2) absence of both medical and surgical treatment before obtaining the tumor sample for high-resolution 1H magic angle spinning (HRMAS) analysis, and 3) tissue samples not contaminated by the histopathological fixing medium.

Eighteen specimens of synchronous, histologically proven liver metastases from 13 patients with small intestine neuroendocrine tumors (SI-NETs) were also collected and analyzed. In addition, 18 and nine samples of respectively normal ileum and normal liver parenchyma were obtained from patients undergoing resection of SI-NETs, isolated or combined with liver metastasectomy. In these cases, the absence of tumoral invasion was histologically verified. The data collected included demographics, preoperative workup data (including clinical, biological and imaging data) and pathological data after surgical resection, including, when available, the degree of parietal infiltration, tumor cellular differentiation and ki-67 index, tumor grade, and the presence of lymphovascular and perineural invasion. The number of primary SI-NETs and the presence of metastatic disease were also investigated. A tumor was defined as “functional” when the patient presented with carcinoid syndrome defined as the coexistence of flushing, diarrhea, and urinary 5-hydroxyindoleacetic acid (5-HIAA) levels higher than 47 μmol/24 h.

This work is part of the CARMeN project (Ethics Committee of Strasbourg, registration number: 100/2003) and tissue samples were obtained from the Tumor Biobank of Strasbourg University Hospitals (CRB, AC 2008-438/DC 2009-1016) and from “Tissu-Tumorothèque Est” (CRB-HCL, Hospices Civils de Lyon Biobank, BB-0033-00046). The research was conducted ethically in accordance with the World Medical Association Declaration of Helsinki. Written informed consent was obtained from all patients included in the present study.

### 4.2. Tissue Sample Preparation

For HRMAS analysis, samples of 15 to 20 mg of tissue were used. Tissue specimens were collected after surgery and snap-frozen in liquid nitrogen before storage at −80 °C. For each sample, the percentage of tumor cells and the percentage of necrosis with regard to the total surface were calculated based on frozen sections using a mirror sample stored in the tissue bank. Samples containing at least 30% tumor cells and less than 50% necrosis were used for the study. Each tissue sample was introduced into a 30 μL disposable insert. 10 μL of D2O were added to the rotor to provide a lock frequency for the nuclear magnetic resonance (NMR) spectrometer. The exact weight of the sample used was determined by weighing the empty insert and the insert containing the tissue sample. The insert was stored at −80 °C and placed in a 4-mm ZrO2 rotor just before the HRMAS analysis.

### 4.3. HRMAS NMR Technical Features

All HRMAS NMR spectra were obtained on a Bruker Advance III 500 spectrometer (Bruker GmbH, Germany) installed at Hautepierre University Hospital, Strasbourg, operating at a proton frequency of 500.13 MHz. A one-dimensional (1D) proton spectrum using a Carr-Purcell-Meiboom-Gill (CPMG) pulse sequence was acquired with an interpulse delay of 285 μs and an acquisition time of 10 min for each tissue sample. The number of loops was set to 328, giving the CPMG pulse train a total length of 93 ms. To confirm resonance assignments in a few representative samples, two-dimensional (2D) heteronuclear experiments (1H–13C) were also recorded immediately after ending the 1D spectra acquisition. Metabolites were assigned using standard metabolite chemical shift tables available in the literature [14].

HRMAS NMR spectra were recorded on a Bruker Advance III 500 spectrometer operating at a proton frequency of 500.13 MHz and equipped with a 4-mm double resonance (1H, 13C) gradient HRMAS probe. A Bruker Cooling Unit was used to regulate the temperature by cooling down the bearing air flowing into the probe. To minimize the effects of tissue degradation, all ex vivo spectra were acquired at a temperature of 4 °C. This value was calibrated exactly using a 100% methanol sample. To keep the rotation sidebands out of the spectral region of interest and to minimize sample degradation, all NMR experiments were conducted on samples spinning at 3502 Hz. For each sample, a one-dimensional proton spectrum using a Carr-Purcell-Meiboom-Gill (CPMG) pulse sequence with presaturation of the water signal was acquired. To eliminate signal losses due to B1 inhomogeneity, the inter-pulse delay between the 180° pulses of the CPMG pulse train was synchronized with the sample and set to 285 µs.

The number of loops was set to 328, thus giving the CPMG pulse train a total length of 93 ms. The parameters for the CPMG experiment were set as follows: sweep width, 14.2 ppm; number of points, 32k; relaxation delay, 2 s; and acquisition time, 2.3 s. A total of 128 FIDs (free induction decay) were acquired resulting in an acquisition time of 10 min. Spectra were referenced by setting the lactate doublet chemical shift to 1.33 ppm. Peaks of interest were automatically defined by an in-house program using MATLAB 7.0 (MathWorks, Natick, MA, USA). In order to confirm resonance assignments, two-dimensional (2D) heteronuclear experiments (1H–13C) were also recorded immediately after the end of 1D spectra acquisition. Because the duration of these experiments was long, they were acquired on only a few samples representative of each class of tissues and exclusively for signal assignment. Significant tissue degradation occurs during this long measurement time; therefore, 1H CPMG experiments were completed before 2D signal assignment experiments. Metabolites were assigned using standard metabolite chemical shift tables available in the literature [14]. Data were zero-filled to a 2k × 1k matrix and weighted with a shifted square sine-bell function before Fourier transformation (FTIR). HRMAS NMR signals were bucketed into integral regions 0.01 ppm wide (ppm range, 8.65–1) using AMIX 3.8 software (Bruker GmbH, Germany) and exported into SIMCA P (version 11.0, Umetrics AB, Umeå, Sweden). To accommodate the influence of metabolite present at both high and low concentrations, without emphasizing spectral noise, unit variance scaling was employed for all analyses. Spectra were referenced for chemical shift according to the lactate peak.

### 4.4. Metabolite Quantification Procedure

Metabolites were quantified by means of a proprietary program based on a custom MATLAB algorithm in a Windows-based environment. The quantification procedure was based on the pulse length-based concentration measurement (PULCON) [38]. Spectra were normalized according to each sample weight and calibrated using the signal intensity of a 19.3 nmol reference solution of lactate, scanned under the same analytical conditions. The peak integral corresponding to each metabolite’s region was normalized to the integral of the entire spectrum. Only well-defined peaks with no overlapping in the 1D CPMG spectra were selected for quantification. Quantification results were expressed as nmol/mg of tissue.

### 4.5. Statistical Analysis

A combination of principal component analysis (PCA) and partial least square discriminant analysis (PLS-DA) was adopted. A PCA was performed to quickly evaluate the quality of the data and to identify possible outliers. Then PLS-DA was employed to optimize the separation between groups. The region between 7.50 and 0.70 ppm of each 1D HRMAS NMR spectrum was automatically bucketed into integral regions of 0.01 ppm, using AMIX 3.9.14 software (Bruker GmbH, Germany). Once the data set was obtained, it was then exported and analyzed into SIMCA P (version 13.0.3, Umetrics AB, Umeå, Sweden). The following two-class models of PLS-DA were built: (i) primary SI-NETs (including all tumors, only grade 1 lesions, or only grade 2 NETs) vs. normal SI tissue, and (ii) primary SI-NETs vs. hepatic NET metastases. To obtain an overview of the data, two three-class PLS-DA models were also tested: (i) grade 1 SI-NETs vs. grade 2 SI-NETs vs. normal SI and (ii) primary SI-NETs vs. hepatic NET metastases vs. normal liver parenchyma. PLS-DA was performed on the spectral buckets corresponding to variable importance for projection (VIP) values ≥1 were selected and labeled VIP. Cross-validation was used in each PLS-DA model to determine the number of components and to avoid overfitting the data. Two measurements of model quality were reported: R2Y and Q2, representing, respectively, the goodness of fit (i.e., data variation) and the goodness of prediction, as estimated by cross-validation [39].

A Spearman correlation test has been conducted to assess the relationship between variables and the Mann-Whitney U test was performed to compare the metabolites’ concentrations. Statistical significance was defined as *p* < 0.05.

The algorithm to determine expected metabolite level alterations (ADEMA) was applied to metabolite quantification values to evaluate the tumoral metabolic network [18]. Several ADEMA models were built according to clinical and pathological variables such as: (a) carcinoid syndrome (presence or absence), (b) metastatic status (presence or absence of lymphatic and/or systemic metastases), (c) primary tumor multiplicity (presence or absence of multiple synchronous SI-NETs), (d) tumor grade, (e) perineural invasion (presence or absence) and angioinvasion (presence or absence) evaluated on the primary SI-NET, and (f) degree of parietal infiltration by the primary tumor (muscle layer/serosa vs. mucosa/submucosa). As previously mentioned, ADEMA evaluates the changes in groups of metabolites, and not one by one between two defined classes of observations (case and control). ADEMA includes the metabolic network topology and uses mutual information to determine whether those metabolites are biomarkers when considered together, and it can predict the expected change in direction per metabolite when the metabolic network topology is considered. The direction of the expected change was obtained by comparing expected levels (carcinoid syndrome or not). The network was constructed using the Kyoto Encyclopedia of Genes and Genomes (KEGG) [40,41] and Selway’s work [42]. An expected metabolite level for cases and controls was obtained per metabolite.

## 5. Conclusions

HRMAS NMR spectroscopy enables metabolomic characterization of intact NET samples and related hepatic metastases. Our results suggest the existence of complex metabolic pathways in NETs, influencing clinical and pathological patterns, and maybe tumor development and evolution. It is also expected that the metabolomic approach, along with the latest technical improvements, will reveal new targets for prognosis assessment and future therapeutic options. Further long-term follow-up prospective studies with stable Isotope Resolved Metabolomics with isotopically-enriched precursors (^13^C-glucose) could have added value to provide a precise cancer profile, define tumor metabolic reprogramming and therapeutic target and develop new therapeutic drugs.

## Figures and Tables

**Table 1:** Results of the algorithm to determine expected metabolite level alterations (ADEMA) network analysis performed on metabolite quantification values assessed from small intestine neuroendocrine tumor (SI-NET) samples by high-resolution 1H magic angle spinning (HRMAS) nuclear magnetic resonance (NMR) spectroscopy. Seven ADEMA models were performed according to patient clinical characteristics and tumor pathological features. ADEMA predicts the expected change in direction per metabolite. An expected metabolite level for cases and controls was obtained per metabolite.

Metabolite	Tumor Grade	Perineural-Invasion	Angio-Invasion	Parietal Infiltration	Tumor Secretion	Metastases at Diagnosis	Primary T Multifocality
	G1 (35) vs. G2 (11)	Yes (13) vs. No (8)	Yes (16) vs. No (8)	Deep (38) vs. Superficial (5)	Yes (13) vs. No (33)	Yes (31) vs. No (15)	Yes (16) vs. No (30)
Taurine	=	up	up	up	=	=	up
Aspartate	up	up	=	down	down	up	up
Serine	up	down	down	down	down	down	down
Acetate	up	up	down	down	down	down	down
NAA	up	down	down	up	up	down	down
Isoleucine	up	down	down	up	down	up	down
Glucose	up	down	=	down	down	down	=
Glycine	down	=	down	down	=	=	=
Valine	up	down	down	=	down	up	down
Lactate	=	up	up	up	up	up	up
Alanine	=	=	down	up	down	up	down
Myoinositol	=	=	=	up	=	=	up
Ascorbate	up	=	=	down	up	up	=
GSH	=	=	=	=	=	=	=
Glutamate	=	=	down	up	=	=	=
Scylloinositol	=	down	=	up	down	=	up
Succinate	up	down	=	=	=	=	up
Fumarate	=	up	down	down	up	down	down
Glutamine	up	=	down	up	up	down	=
Arginine	up	=	down	up	up	up	up
Creatine	up	=	down	down	down	up	up
Ethalonamine	up	down	down	=	=	up	=
Choline	=	up	up	=	up	up	up
GPC	=	up	up	=	=	up	up
PC	=	=	=	down	up	=	up
Tyrosine	up	down	down	up	up	down	down
Tryptophane	up	up	up	up	=	up	=

Metabolite: detected and quantified metabolites from tissue specimens; Up: metabolites that are predicted to increase (red); Down: metabolites that are predicted to decrease (blue); =: metabolites that are expected to stay the same; NAA: N Acetyl Aspartate; GSH: Glutathione; GPC: Glycerophosphocholine; PC: Phosphorylcholine; Deep parietal infiltration: tumoral invasion of muscle layer and serosa; Superficial parietal infiltration: tumoral infiltration limited to mucosa and sub mucosa. Number of analyzed samples is reported in brackets for each ADEMA model.

## Supplementary Materials

The following are available online at https://www.mdpi.com/2218-1989/9/12/300/s1, Table S1: 1H NMR resonance assignments of 27 metabolites identified and quantified within the range of 7.50–0.70 ppm in the 94 analyzed tissular samples, including 46 primary NETs, 18 hepatic NET metastases and 30 specimens of normal small-intestine and liver parenchyma. Spectra were referenced by setting the lactate doublet chemical shift to 1.33 ppm. Metabolites were assigned using standard available metabolite chemical shift tables (14).
