# Supplementary material for: Metabolomics of Small Intestine Neuroendocrine Tumors and Related Hepatic Metastases
Source: Metabolites. 2019 Dec 11;9(12):300. doi: 10.3390/metabo9120300 (PMC6950539; doi:10.3390/metabo9120300)
Supplement: Supplementary file 1 [file metabolites-09-00300-s001.pdf]

Table 1

N °	Metabolite	Group	^{1}H chemical shift (ppm)
1	Taurine	$-CH_2-NH_3^+$	3.27
		$-CH_2-SO_3^-$	3.42
2	Aspartate	βCH_2(u)	2.69
		βCH_2(d)	2.81
		αCH	3.89
3	Serine	αCH	3.84
		βCH_2(u)	3.94
		βCH_2(d)	3.99
4	Acetate	CH_3	1.92
5	N Acetyl Aspartate (NAA)	CH_3	2.02
6	Isoleucine	δCH_3	0.94
		γCH_3	1.01
		γCH_2	1.27
		αCH	3.68
7	β-Glucose	C4H	3.41
		C3H, C5H	3.48
		C6H(u)	3.76
		C6H(d)	3.89
		C1H	4.65
8	Glycine	CH_2	3.56
9	Valine	γCH_3	0.99
		$\gamma' CH_3$	1.04
		βCH	2.28
10	Lactate	CH_3	1.33
		CH	4.12
11	Alanine	βCH_3	1.48
		αCH	3.78
12	myo-Inositol	C5H	3.27
		C1H, C3H	3.53
		C4H, C6H	3.62
		C2H	4.05
13	Ascorbate	-CHOH-	4.02
		C4H	4.52
14	Glutathione (GSH)	CH_2-CONH	2.56
		CH_2-SH	2.95
		CH-NH_2	3.79
15	Glutamate	βCH_2	2.09
		γCH_2	2.34
		αCH	3.76
16	scyllo-Inositol	all Hs	3.34
17	Succinate	(α,β CH2)	2.41
18	Fumarate	all CH	6.52
19	Glutamine	βCH_2	2.14
		γCH_2	2.45
		αCH_2	3.78
20	Arginine	γCH_2(u)	1.64
		γCH_2(d)	1.72
		βCH_2	1.92
		δCH_2	3.25
		αCH	3.78
21	Creatine	CH_3	3.03
		CH_2	3.94
22	Ethanolamine	$-CH_2-NH_3^+$	3.14
		CH_2OH	3.82
23	Choline	$-N^+-(CH_3)_3$	3.22
		βCH_2	3.54
		αCH	4.07
24	Glycerophosphocholine	$-N^+-(CH_3)_3$	3.24
		αCH_2-OH	3.65
		βCH_2-OH and $-CH_2-N^+$	3.70
		CH_2-PO_4 and CHOH	3.92
		CH_2-PO_4	4.33
25	Phosphorylcholine	$-N^+-(CH_3)_3$	3.23
		βCH_2	3.61
		αCH	4.16
26	Tyrosine	βCH_2(u)	3.06
		βCH_2(d)	3.20
		αCH	3.94
		orthoCH	6.89
		metaCH	7.18
27	Tryptophan	βCH_2(up)	3.31
		βCH_2(down)	3.49
		αCH	4.06
		CH-NH	7.32
